# Elucidating the progress and impact of ferroptosis in hemorrhagic stroke

**DOI:** 10.3389/fncel.2022.1067570

**Published:** 2023-01-11

**Authors:** Feixia Pan, Weize Xu, Jieying Ding, Chencen Wang

**Affiliations:** ^1^Department of Cardiac Surgery, The Children’s Hospital, Zhejiang University School of Medicine, National Clinical Research Center for Child Health, Hangzhou, China; ^2^Department of Pharmacy, The Children’s Hospital, Zhejiang University School of Medicine, National Clinical Research Center for Child Health, Hangzhou, China; ^3^Department of Pediatrics, The First People’s Hospital of Yongkang Affiliated to Hangzhou Medical College, Jinhua, China

**Keywords:** stroke, intracerebral hemorrhage, subarachnoid hemorrhage, ferroptosis, target, lipid peroxidation

## Abstract

Hemorrhagic stroke is a devastating cerebrovascular disease with high morbidity and mortality, for which effective therapies are currently unavailable. Based on different bleeding sites, hemorrhagic stroke can be generally divided into intracerebral hemorrhage (ICH) and subarachnoid hemorrhage (SAH), whose pathogenesis share some similarity. Ferroptosis is a recently defined programmed cell deaths (PCDs), which is a critical supplement to the hypothesis on the mechanism of nervous system injury after hemorrhagic stroke. Ferroptosis is characterized by distinctive morphological changes of mitochondria and iron-dependent accumulation of lipid peroxides. Moreover, scientists have successfully demonstrated the involvement of ferroptosis in animal models of ICH and SAH, indicating that ferroptosis is a promising target for hemorrhagic stroke therapy. However, the studies on ferroptosis still faces a serious of technical and theoretical challenges. This review systematically elaborates the role of ferroptosis in the pathogenesis of hemorrhagic stroke and puts forward some opinions on the dilemma of ferroptosis research.

## 1. Introduction

Hemorrhagic stroke is an acute disease caused by the sudden rupture of a specific blood vessel in the brain. It refers to two main diseases based on bleeding sites: bleeding within brain parenchyma termed intracerebral hemorrhage (ICH) or within the subarachnoid space called subarachnoid hemorrhage (SAH) (Doria and Forgacs, 2019; Fang et al., 2020). Hemorrhagic stroke accounts for 10–20% of all strokes and is responsible for more than 40% of stroke-related deaths caused by severe primary and complicated secondary brain injury (Doria and Forgacs, 2019). Currently, the treatment of hemorrhagic stroke has not been well-established. Apart from the recognized supportive care, the efficacy of antihypertensive therapy, complication prevention, surgery, and hemostatic therapy to reduce primary brain injury remain controversial (Grysiewicz et al., 2008; Xu Y. et al., 2022). Moreover, despite the considerable pre-clinical efficacy of several pharmacological agents targeting pathological events of hemorrhagic stroke, they failed to perform in clinical trials as factors such as the short half-life periods of agents and the role of the blood-brain barrier (BBB) complicated the scenario (Grysiewicz et al., 2008). Further efforts are necessary to develop potential drugs against novel pathological targets to facilitate functional recovery for hemorrhagic stroke patients. Numerous pathological processes during the secondary brain injury lead to the death of neuronal cells after hemorrhagic stroke. In addition to extensively researched apoptosis, many other modes of cell death, including ferroptosis, have been explored in ICH and SAH animal models in recent years (Fang et al., 2020; Shen et al., 2020).

Based on previous studies, Dixon et al. (2012), defined a mode of programmed cell death (PCD) caused by iron overload-dependent accumulation of lethal lipid peroxides as “ferroptosis” (Dixon et al., 2012). Morphologically, ferroptosis is characterized by mitochondrial shrinkage, loss of mitochondrial cristae, and increased mitochondrial membrane density. Biochemically, the ferroptosis-mediated changes include defective glutathione (GSH) activity, excessive accumulation of lethal lipid reactive oxygen species (ROS) on the plasma membrane, the inactivation of glutathione peroxidase 4 (GPX4), and the abnormal activation of some lipid metabolism regulatory molecules (Dixon et al., 2012; Stockwell et al., 2017; Wang et al., 2021). Before the concept was proposed, most of these ferroptosis-associated biochemical changes were detected in animal models of hemorrhagic stroke (Chang et al., 2014; Gao et al., 2020; Cao et al., 2021; Duan et al., 2021; Qu et al., 2021). Several recent studies have systematically examined these markers and monitored key targets regulating ferroptosis, confirming the presence of ferroptosis in hemorrhagic stroke diseases (Alim et al., 2019; Cao et al., 2021). More importantly, many studies demonstrated that inhibition or negative modulation of ferroptosis in neuronal cells was promising as a potential therapy for treating hemorrhagic stroke (Chang et al., 2014; Shao et al., 2019; Qu et al., 2021; Ren et al., 2022). However, limited by the objective reasons of experimental technological challenges, many critical issues still remain to be discussed and resolved. Therefore, in this review, we explored the current status of ferroptosis research advances in animal models of SAH and ICH. We also summarized the findings of the studies on the drugs or inhibitors targeting ferroptosis and further discussed their feasibility as potential therapies for treating hemorrhagic stroke.

Search strategy: A systematic search was implemented on two databases PubMed and Web of Science. The overall retrieval terms are “intracerebral hemorrhage AND ferroptosis,” and “subarachnoid hemorrhage AND ferroptosis.” In the part of Iron overload, retrieval terms “(intracerebral hemorrhage or subarachnoid hemorrhage) AND (iron OR iron disorder OR iron overload)” were used. In the part of Lipid peroxidation, retrieval terms “(intracerebral hemorrhage or subarachnoid hemorrhage) AND (lipid peroxidation OR reactive oxygen species)” were used. In the part of antioxidant system Lipid peroxidation, retrieval terms “(intracerebral hemorrhage or subarachnoid hemorrhage) AND (FSP1/CoQ10 OR GSH/GPX4)” were used. All the references were loaded into Zotero (version 5.0.96) and combined to exclude duplicate and triplicate.

## 2. Essential characteristics and mechanisms of ferroptosis

**FIGURE 1:**
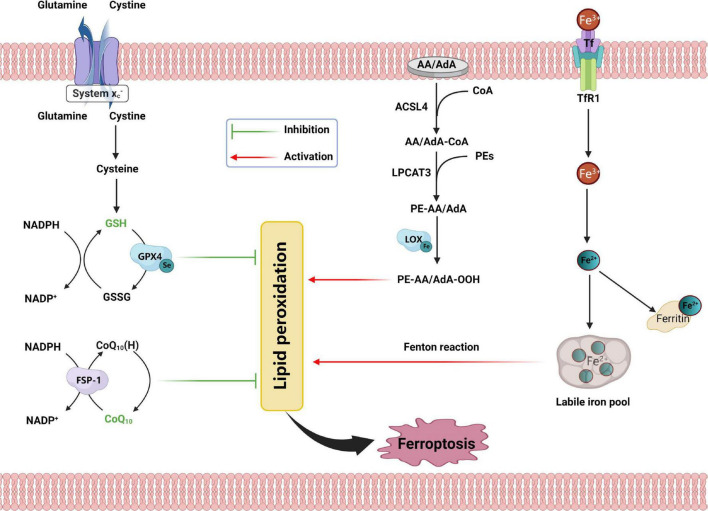
Schematic representation for the mechanism of ferroptosis, including antioxidant systems, iron disorder, and lipid peroxidation. SLC7A11, solute carrier family 7 member 11; SLC3A2, solute carrier family 3 member 2; GSH, glutathione; GSSG, oxidized glutathione; GPX4, glutathione peroxidase 4; NADPH, nicotinamide adenine dinucleotide phosphate; GPX4, glutathione peroxidase 4; CoQ10, coenzyme Q10; FSP1, ferroptosis suppressor protein 1; AA, arachidonic acid; AdA, adrenic acid; CoA, coenzyme A; ACSL4, acyl-CoA synthetase long-chain family member 4; AA/AdA-CoA, adrenic acid/arachidonic acid-CoA; PEs, phosphatidylethanolamines; LPCAT3, lysophosphatidylcholine acyltransferase 3; PE-AA/AdA, phosphatidylethanolamine-adrenic acid/arachidonic acid; LOX, lipoxygenase; PE-AA/AdA-OOH, phosphatidylethanolamine- adrenic acid/arachidonic acid bis/trioxide; Tf, transferrin; TFR1, transferrin receptor 1.

A decade before the term “ferroptosis” was defined, the basic features of ferroptosis were identified in quick succession (Figure 1). The two most used drugs for inducing ferroptosis, erastin, and Ras-selective lethal small molecules 3 (RSL3), were identified by the same research team in 2003 and 2008, respectively (Dolma et al., 2003; Yang and Stockwell, 2008). This group reported that they induce a form of PCD different from apoptosis. It was shown that iron chelator (deferoxamine) and certain antioxidants explicitly inhibit this type of cell death. Based on previous studies, Dixon et al. (2012) named this form of PCD as “ferroptosis” in 2012 and systematically described its biological, biochemical, and genetic characteristics. After about 5 years, studies from several different laboratories comprehensively summarized the morphological features of ferroptosis, which manifest as damaged mitochondria including mitochondrial shrinkage, reduced amounts of cristae, and outer membrane rupturing (Kagan et al., 2017). Mitochondrial structure monitoring by transmission electron microscope (TEM) is crucial for determining the occurrence of ferroptosis in the cells (Dixon et al., 2012). From the biochemical standpoint, ferroptosis involves a complex molecular biological network leading to the overproduction and accumulation of lipid peroxidation products and disrupting the antioxidant systems (Dixon et al., 2012; Stockwell et al., 2017). These include landmarks that are widely recognized by researchers like GPX4, SLC7A11 (xCT), and acyl-CoA synthetase long-chain family member 4 (ACSL4). Moreover, there are also many new targets that have been successively proven to be related to ferroptosis in recent years (Lei et al., 2022). In this review, we attempt to make a succinct summary of all such information below.

### 2.1. Disorders of iron metabolism

Ferroptosis is inhibited by iron chelators, which have led scientists to initially name it a new PCD with a root “ferr-” representing the then-popular belief that iron is indispensable in this process. However, this led to a misconception for future research, whereby people believed that ferroptosis must be accompanied by the disturbance of iron metabolism or even iron overload. Many subsequent landmark studies did not pay attention to iron metabolism-related factors because lethal lipid-related ROS and disruption of the antioxidant system are the key factors mediating ferroptosis (Li and Li, 2020). Moreover, the construction of many *in vitro* ferroptosis models does not depend on any form of iron (Ding et al., 2020; Liu et al., 2020). Despite no apparent link between iron overload and ferroptosis, substantial attention is warranted to investigate the possibility of ferroptosis during iron overload.

Under normal circumstances, ferrous (Fe2^+^) ions from physiological or pathological origin get oxidized to ferric (Fe3^+^) ions. They combine with free transferrin (TF), which is recognized by membrane protein transferrin receptor 1 (TFR1). Then the TF/TFR1 complex will be taken up in a manner of endocytosis. Because of the acidic environment in endosome, Fe3^+^ will immediately be reduced to Fe2^+^ by ferric reductase sixtransmembrane epithelial antigen of prostate 3 (STEAP3) after being released from TF (Louandre et al., 2013). After that, divalent metal transporter 1 (DMT1) transports Fe2^+^ into the labile iron pool in the cytoplasm (Katsarou and Pantopoulos, 2020). It is noteworthy that ferroportin-1 (FPN1) on the cell membrane is currently the only known molecule to actively transport Fe2^+^ from the intracellular to the extracellular region, maintaining the stability of the intracellular iron pool (Troadec et al., 2010). The change in the function or content of the above-mentioned iron regulatory molecules may cause the iron overload in cells, inducing ferroptosis. A recent study found that activation of nuclear receptor coactivator 4 (NCOA4) destabilizes ferritin in an autophagy-dependent manner, increasing the labile iron level and promoting ferroptosis (Zhang Y. et al., 2021).

### 2.2. Excessive accumulation of lipid peroxides

Lipid peroxides not removed in time are the most direct executors of ferroptosis. The following three pathways generate the free radical reactions mediating the formation of lethal lipid peroxides: (i) non-enzymatic Fenton reaction; (ii) iron-catalyzed auto-oxidation, and (iii) oxidase- and esterase-mediated peroxidation of polyunsaturated fatty acids (PUFA) (Yang and Stockwell, 2016; Li and Li, 2020). The polyolefinic structure of PUFAs, especially arachidonic acid (AA) and adrenic acid (ADA), makes them susceptible to oxidation to form lipid peroxides. However, studies show that excess peroxidation of PUFAs (AA/ADA-OOH) does not explicitly indicate ferroptosis. AA/ADA must be catalyzed by acyl-CoA synthetase long-chain family member 4 (ACSL4) to form AA/ADA-CoA, followed by esterification into membrane phospholipids before becoming a crucial precursor of lethal lipid-related ROS (Kagan et al., 2017; Wenzel et al., 2017). Two independent groups have implicated using lipid metabolomics screening that the product of phosphatidylethanolamines (PEs)-AA/ADA, namely, PEs-AA/ADA-OOH, oxidized by lipoxygenase, as the critical substance mediating ferroptosis. There are numerous ways of catalyzing PEs-AA/ADA to form peroxides (Kagan et al., 2017; Wenzel et al., 2017). It has been shown that the rate-limiting steps in acetylation and esterification make ACSL4 and lysophosphatidylcholine acyltransferase 3 (LPCAT3) the critical promoters in the regulation of ferroptosis (Feng et al., 2022). Multiple studies have confirmed the roles of these enzymes. Numerous recent studies have indicated how inhibition of ACSL4 and LPCAT3 might be promising therapeutic strategies for treating ferroptosis-related injuries (Cui et al., 2021a; Sha et al., 2021; Reed et al., 2022). However, how PEs-AA/ADA-OOH relay the signals for ferroptosis remains to be interpreted. Plausible underlying mechanisms include binding excess lipid peroxides to the membrane, causing lethal changes in membrane permeability and ion concentrations. It is also believed that the downstream products of these lipid peroxides, like malondialdehyde (MDA) and 4-hydroxy-nonenal (4-HNE), exert ferroptotic damage by degrading proteins or nucleic acids (Liu et al., 2020, 2021). Many studies have implicated MDA and 4-HNE as markers of ferroptosis.

### 2.3. GSH/GPX4 antioxidant system

GPX4 is an important peroxide-decomposing enzyme ubiquitously present in the body. GSH can complete the conversion of lipid peroxides to non-toxic lipids (Fraternale et al., 2009). This property protects cell membranes made up of large lipid molecules from peroxides interference and damage. GSH/GPX4 antioxidant system is one of the most crucial endogenous factors against ferroptosis (Li F. et al., 2022). GSH is synthesized by three amino acids: glutamate, cysteine, and glycine. Cysteine, mainly derived from extracellular cystine after entering the cell and being reduced, is present in the lowest concentration in the cell, making it the critical rate-limiting factor for the *de novo* synthesis of GSH. The cystine/glutamate antiporter (system X_*C*_^–^) on the cell membrane transports the extracellular cysteine into the cell in a 1:1 form while transporting out the intracellular glutamate (Yuan Y. et al., 2022). The system X_*C*_^–^ on the cell membrane is a heterodimer consisting of the substrate-specific subunit SLC7A11 (xCT) and the regulatory subunit SLC3A2 (Koppula et al., 2021). GPX4 converts the reduced form of GSH into oxidized glutathione (GSSG) and promotes the reduction reaction from lipid peroxides (L–OOH) to corresponding alcohols (L–OH) (Li F. et al., 2022).

In many pathological conditions or artificial interventions, increased extracellular glutamate and disrupted xCT structure or function lead to intracellular GSH depletion, thereby inducing ferroptosis (Chen et al., 2017; Yadav et al., 2021). Meanwhile, the down-regulation or functional disruption of GPX is also essential for promoting ferroptosis. The most used inducers of ferroptosis, erastin, and RSL3, are inhibitors of system X_*C*_^–^ and direct inhibitors of GPX4, respectively (Stockwell et al., 2017). In addition, there are many compounds that can induce ferroptosis by inhibiting GPX4, like DPI7 and DPI10 in a directing binding manner and FIN56 in a form that induces GPX4 degradation (Mou et al., 2019; Sun et al., 2021). On the other hand, selenium promotes GPX4 transcription and prevents it from irreversible degradation, thereby inhibiting ferroptosis (Alim et al., 2019; Yao et al., 2021). Increasing evidence delineate the role of the GSH/GPX4 antioxidant system in the mechanism of ferroptosis.

### 2.4. FSP1/CoQ10 and its related antioxidant system

The ferroptosis suppressor protein 1 (FSP1)/coenzyme Q10 (CoQ10) pathway is another efficient endogenous antioxidant system independent of GPX4/GSH system, that critically regulates cellular sensitivity toward ferroptosis. FSP1, alternatively known as apoptosis-inducing factor mitochondria 2 (AIFM2), has significantly high homology with apoptosis-inducing factor (AIF) (Miriyala et al., 2016). The name and homology details of AIFM2 indicate how it was identified by its function to induce apoptosis by releasing cytochrome c and caspase 9 (Miriyala et al., 2016). However, a recent study found that AIFM2 had a marked inhibitory effect on GPX4 deficiency that leads to ferroptosis, renamed “FSP1” (Doll et al., 2019). The lethal lipid peroxidation event in ferroptosis mainly starts at the plasma membrane, leading to the transfer of FSP1 by myristoylation for subsequent free radical scavenging (Doll et al., 2019). FSP1 uses the reducing equivalents of nicotinamide adenine dinucleotide phosphate [NAD(P)H] to reduce CoQ10 to ubiquinol, which acts as a lipophilic radical-trapping antioxidant (RTA) to remove lipid peroxides from phospholipid bilayers (Bersuker et al., 2019).

NAD(P)H is also indispensable for reducing GSSG to GSH. Therefore, involved in two important antioxidant systems, the level of NAD(P)H have also been indicated by several studies as a predictor of cellular susceptibility to ferroptosis (Bersuker et al., 2019). This molecular crosstalk means that the intracellular defenses against lipid peroxides are not dependent on a single system. FSP1 was also recently reported to inhibit ferroptosis in endosomal required transport (ESCRT)-III-dependent manner (Dai et al., 2020). ESCRT-III is a membrane-associated protein complex enhancing plasma membrane repair by activating subtypes of charged multivesicular body proteins (Dai et al., 2020). In addition, tetrahydrobiopterin (BH4) and its synthetic rate-limiting enzyme Guanosine triphosphate cyclohydrolase 1 (GCH1) are involved in the ferroptosis-resistant oxidative network by regulating CoQ10 synthesis (Mishima and Conrad, 2022). However, more investigation is required to confirm these systems’ effectiveness and underlying mechanisms.

## 3. The role of ferroptosis in hemorrhagic stroke

According to the bleeding site, a hemorrhagic stroke can be divided into two subtypes, ICH and SAH (Doria and Forgacs, 2019). Their pathological mechanisms occur in two steps and involve multiple similarities. The first stage involves primary brain injury dominated by mechanical damage caused by the traction of the hematoma, accompanied by increased intracranial pressure and secondary cerebral infarction (Magid-Bernstein et al., 2022). The second stage consists of pathophysiological events caused by blood components and their metabolites, including BBB destruction, neural excitatory events, ion disturbances, oxidative stress, neuroinflammation, cell death, etc (Magid-Bernstein et al., 2022). In SAH, early brain injury (EBI) is crucial, indicating the pathological damage within 3 days after SAH (Kusaka et al., 2004; Fujii et al., 2013). The recovery degree of the pathological damage during EBI will substantially impact the recovery of subsequent neurological functions.

Several animal models were adopted for hemorrhagic stroke research. For ICH, collagenase injection or autologous blood injection at the basal ganglia are two common modeling methods (Krafft et al., 2012; Lei et al., 2014). The former causes cerebral hemorrhage by decomposing collagen on the cell matrix and vascular basement membrane, which has the advantage of high efficiency and convenience. While the latter can better simulate the pathophysiological changes of patients with cerebral hemorrhage. For SAH, the endovascular perforation model is widely used because it can simulate SAH caused by rupture of aneurysm (Du et al., 2016). These models can cause a large amount of instantaneous hemorrhage in specific regions of the brain, and are valuable experimental models of hemorrhagic stroke in preclinical research.

Degradation products of hemoglobin (ferrous iron and heme) play an essential initiating role in the induction of these pathophysiological events. Among them, the abnormal accumulation of iron ions leads to severe tissue damage (Gaasch et al., 2007). It generates a large amount of ROS, providing a solid foundation for ferroptosis to set in Gaasch et al. (2007). This validates the hypothesis that ferroptosis plays a critical pathological role after a hemorrhagic stroke. A series of subsequent studies also confirmed the occurrence of ferroptosis in animal and *in vitro* models of ICH and SAH (Karuppagounder et al., 2018; Chen et al., 2019; Cao et al., 2021; Qu et al., 2021). Li et al. (2018) first observed specific mitochondrial morphology of ferroptosis in perihematoma neurons in a collagenase-induced mouse ICH model. Then in 2020, ferroptotic mitochondria were first observed in temporal cortical neurons in a mouse model of SAH (Li Y. et al., 2021). The TEM pictures of mitochondria provide strong evidence supporting ferroptosis. In addition, the down-regulation of the antioxidant molecule GPX4 and upregulation of ACSL4 and lipid peroxides were also validated in these two models by various groups (Zhang et al., 2018; Gao et al., 2020; Jin et al., 2021; Qu et al., 2021). These results demonstrate how ferroptosis, an essential complement to other PCDs, plays a vital role in hemorrhagic stroke. Moreover, two critical inhibitors of ferroptosis, Ferrostatin-1 (fer-1) and Liproxstatin-1 (Lip-1), inhibited neuronal ferroptosis and neuroinflammation in animal models of hemorrhagic stroke, reducing neurological impairment (Li et al., 2017a; Zhang et al., 2018; Cao et al., 2021). In addition, various chemical agents and natural medicines improve neurological damage in animals with ICH or SAH by inhibiting ferroptosis, suggesting ferroptosis as a promising potential target for therapeutic strategies for hemorrhagic stroke (Li et al., 2019; Duan et al., 2021; Gao et al., 2022).

However, the knowledge about ferroptosis in hemorrhagic stroke is still minimal. To achieve clinical translation, sufficient preclinical experiments must identify effective therapeutic agents and the exact molecular mechanisms.

## 4. Crosstalk between ferroptosis and other PCDs in hemorrhagic stroke

In addition to ferroptosis, other PCDs such as apoptosis, pyroptosis, necroptosis, and autophagy are also widely studied in the field of hemorrhagic stroke. There are also various crosstalk between ferroptosis and these PCDs. Apoptosis is the most well-studied forms of PCD in hemorrhagic stroke, which mainly induced by two pathways: extrinsic and intrinsic pathways (Sekerdag et al., 2018). The characteristic features of apoptotic neuron include chromatin condensation, nuclear shrinkage, and DNA fragmentation, which is completely different from ferroptosis (Weiland et al., 2019). Hence, apoptosis, being considered as the main form of neuronal death after hemorrhagic stroke, is mainly studied as an exclusionary form in iron death related studies. A recent study suggests that there may be a certain correlation between ferroptosis and necroptosis through NADPH (Hou et al., 2019). Since autophagy can regulate intracellular iron homeostasis and ROS synthesis, current research indicates that many ferroptosis are autophagic dependent (Ma et al., 2022; Tao et al., 2022). The latest research of Tao et al. (2022) indicates that S100A8 can mediate autophagy-dependent ferroptosis of microglia after SAH. In addition, mitophagy has also been reported to mediate ferroptosis (Li J. et al., 2022), which has not been verified in hemorrhagic stroke, indicating it may be a valuable research direction in the future.

## 5. The research status in ferroptosis in hemorrhagic stroke

Subarachnoid hemorrhage (SAH) and intracerebral hemorrhage (ICH) share many pathological similarities, and similar hypotheses have been formed to identify the mechanism of ferroptosis in these two hemorrhagic strokes. However, to date, the underlying mechanisms of ferroptosis have not been understood to the same extent in ICH and SAH, triggered by differential research investments. Because the incidence of SAH is much lower than ICH worldwide, the progress of basic research in decoding the mechanisms of ferroptosis is also relatively inadequate in SAH (Doria and Forgacs, 2019). However, the advances in research to decode ferroptosis mechanisms in SAH have not been summarized. Therefore, in this part, we will systematically review the research progress in understanding ferroptosis mechanisms in ICH and SAH.

### 5.1. Iron overload in ICH

The direct accumulation of blood components in the injured area is a critical pathogenic symptom of hemorrhagic disease. Therefore, since the discovery of ICH, researchers have long been critically concerned about the disturbance of iron metabolism (Uselis, 1970). As early as 2004, a study identified iron deposition in the basal ganglia in a rat model of ICH (Nakamura et al., 2004). This deposited iron is mainly derived from the lysed red blood cell hemoglobin, which is degraded into heme and free iron. In addition, Nakamura et al. (2005) indicated that holo-transferrin interacts with thrombin to exacerbate brain damage upon the onset of ICH. Iron accumulation and ferritin upregulation also cause long-term neurological impairment in animal models of ICH (Nakamura et al., 2005). Deferoxamine (DFO) is a drug that eliminates iron accumulation and has been validated by multiple research groups in various animal models. Its therapeutic mechanism has been hypothesized to involve DNA oxidative damage repair, neuronal apoptosis inhibition, and brain edema reduction (Farr and Xiong, 2021). In addition, Zhao et al. (2011) reported another potent iron chelator, minocycline, which alleviates neuronal apoptosis after ICH by inhibiting the pathways that upregulate iron handling proteins of the brain. DFO and minocycline have been selected for clinical trials because of their excellent efficacy in animal experiments. Unfortunately, no drug targeting iron chelation has successfully achieved clinical translation (Selim et al., 2011). Moreover, the specific mechanism underlying cellular damage due to ROS generation by iron overload has not been elucidated.

Introducing the concept of ferroptosis provides insights into a new pathogenic mechanism of iron-mediated diseases, encouraging researchers to delve deep into it. Hemorrhage leads to iron deposition and regulates iron’s entry and exit pathways by several vital molecules, including TF, TFR1, DMT1, and FPN1 (Vogt et al., 2021). Hepcidin has received more attention in recent years due to its ability to change intracellular iron content by regulating the expression of TF, TFR1, DMT1, and FPN1 (Camaschella et al., 2020). TF, TFR1, and DMT1 are responsible for iron uptake, while FPN1 is the only regulatory molecule aiding in the transport of iron out of the cells (Vogt et al., 2021). Yang et al. (2021) have demonstrated how ICH mice exhibited less iron overload, brain edema, and neuronal damage after adeno-associated virus-mediated overexpression of hepcidin. Down-regulating TF and TFR1 mainly achieve this effect after ICH. In recent years, many new iron chelators have been tested on animal models of ICH and achieved considerable preclinical efficacy. VK-28, Deferiprone (DFP), and Deferasirox (DFX) are some representative drugs (Wang et al., 2016; Li et al., 2017b; Imai et al., 2021). However, it is worth pointing out that although most studies of ferroptosis in ICH involve some iron deposition, few studies have directly explored the association between iron deposition and ferroptosis. We believe this action stems from the lack of a specific mechanism of iron-mediated ferroptosis.

The STICH II trial showed that withdrawal after open surgery didn’t significantly reduce the mortality of cerebral hemorrhage compared with the initial conservative treatment (Mendelow et al., 2013). However, the subgroup of MISTIE III trial showed that compared with the conservative treatment group, the patients whose blood clot size decreased less than 15 ml after minimally invasive surgery live with better neurological recovery (Hanley et al., 2019). In addition to iron chelating agent drugs, surgical removal of hematoma or endogenous phagocytosis of ectopic erythrocyte from the source to reduce the degradation products of erythrocyte are effective treatments for hemorrhagic stroke (Zheng et al., 2022). The phagocytosis of erythrocytes is mainly completed by microglia and macrophages, which are regulated by several signal pathways. Peroxisome proliferator-activated receptor γ (PPAR-γ) is one of the most widely studied regulatory molecules, and its activation can promote the phagocytosis of microglia on hematoma. Drugs including monascin, simvastatin, wogonin can activate PPAR-γ, thus promoting phagocytosis (Wang et al., 2018; Fu et al., 2020; Zhuang et al., 2021). In addition, the activation of nuclear factor erythroid 2-related factor2 (Nrf2) pathway has also been proved to promote hematoma phagocytosis (Zheng et al., 2022). Xu C. et al. (2022) demonstrated that CD47 also plays an important role in the process of hematoma phagocytosis in the SAH model.

### 5.2. Iron overload in SAH

Before the concept of early brain injury was proposed, the academic research on the pathogenesis of SAH mainly focused on secondary ischemia caused by vasospasm (Lin et al., 2004). In 1988, the non-glucocorticoid 21-aminosteroid U74006F, an inhibitor of iron-dependent lipid peroxidation, alleviated the acutely progressive hypoperfusion state in a cat SAH model (Hall and Travis, 1988). In the early 1990s, Utkan et al. (1996) demonstrated for the first time that the iron chelator DFO could prevent SAH-induced vasospasm in a rabbit model (Utkan et al., 1996). They also hypothesized that the underlying mechanism might involve the inhibition of iron-induced ROS and lipid peroxides (Utkan et al., 1996). Subsequent studies have repeatedly demonstrated the reduction of vasospasm by iron chelation (Luo et al., 1995). Smith et al. (1996) primarily focused on iron-induced neuronal damage. They elucidated how a lipid peroxidation inhibitor, tirilazad mesylate (U-74006F), and its metabolites attenuated early BBB damage and attenuated iron-induced neuronal lipid peroxidative damage. This may be the earliest proof of the underlying mechanism of ferroptosis in hemorrhagic stroke.

Similar to studies on ICH, around 2004, researchers systematically studied the metabolism and role of hemoglobin, heme, and iron in SAH and also pointed out the predictive effect of serum ferritin content on the severity of SAH (O’Connell and Watson, 2003; Suzuki et al., 2003). In addition, the up-regulation of TF and TFR1 and ferritin in brain tissue was also verified by Lee et al. (2010) in a rat model of SAH. Their team also showed that iron overload in the acute phase of SAH leads to oxidative stress and neuronal damage, which can be reversed by the application of DFO (Lee et al., 2010). However, since the advent of the concept of ferroptosis, few studies have systematically verified the association between iron overload and cellular ferroptosis in SAH models. Moreover, no study has investigated the neurological rescue effect of various iron chelators achieved by inhibiting ferroptosis. However, many studies have reported a positive correlation between iron accumulation and ferroptosis in ischemic stroke. One study indicated that tau-mediated iron export could reduce ferroptosis after ischemic stroke (Tuo et al., 2017). Mitochondrial ferritin has also been upregulated in the brain of mice with ischemic stroke upon FPN1 upregulation to attenuate ferroptosis and achieve neuroprotective effects (Wang P. et al., 2022). Recently, Zhang H. et al. (2021) pointed out that hepcidin can promote ferroptosis after SAH by regulating iron metabolism, and this effect may be performed by upregulating DMT1. Their research on iron metabolism regulation lacks comprehensiveness, warranting more in-depth studies to explore the correlation between iron overload and ferroptosis.

### 5.3. Lipid peroxidation in ICH

Oxidative stress is essential in ICH pathogenesis, primarily mediated by excessive accumulation of ROS and reactive nitrogen species (RNS) (Li et al., 2011). ROS is a group of partially reduced oxygen-containing molecules or radicals, and its simple form mainly includes hydrogen peroxide (H_2_O_2_) and some radicals such as hydroxyl radical (⋅OH) and superoxide anion (O^2–^) (Zhu et al., 2021). Lipid peroxidation involves ROS-mediated electron loss, forming various reactive intermediates. Before introducing the concept of ferroptosis, numerous studies demonstrated how ROS and end-products of lipid peroxidation like MDA damage proteins and DNA, resulting in cell injury (Wang et al., 2011). Some classic antioxidant drugs, such as metformin and melatonin, provide neuroprotective effects to ICH mice by reducing ROS and MDA levels (Lekic et al., 2010; Wu et al., 2016). However, the mechanisms underlying the pathogenicity of lipid peroxides remain unclear. The synthesis of lipid peroxides relies on several specific regulatory processes, which are thought to be critical factors regulating the initiation of ICH ferroptosis.

The high level of PUFAs in the brain is beneficial for inducing ferroptosis signaling in ICH. 20-hydroxyeicosatetraenoic acid (20-HETE), a metabolite of ADA, was recently shown to be involved in neuronal ferroptosis upon ICH induction in mice (Han et al., 2021). And reduction of its production with the specific inhibitor HET0016 significantly inhibited lipid peroxidation levels and cell death in an *in vitro* model of ICH (Han et al., 2021). This significantly contributed to research on lipid peroxidation in ferroptosis after ICH due to limited previous research exploring this direct relationship. Previous studies indicated that N-acetylcysteine could neutralize the peroxidative toxicity of arachidonic acid catalyzed by 5-lipoxygenase to inhibit ferroptosis after ICH (Karuppagounder et al., 2018). Moreover, baicalin, a non-specific inhibitor of 12/15-lipoxygenase, significantly increases the detection indices related to ferroptosis after ICH (Duan et al., 2021), suggesting that 12/15-lipoxygenase may play a critical role in the process of ferroptosis after ICH.

ACSL4 has also been implicated in the process of ferroptosis in ICH by two recent independent studies (Chen et al., 2021; Jin et al., 2021). It is highly expressed in the brain tissue surrounding the hematoma of ICH mice. Moreover, ACSL4 can also be regulated by long non-coding RNAs (LncRNAs). LncRNA H19 positively regulates ACSL4 expression upon ICH onset (Chen et al., 2021). Another study pointed out that LncRNA HOX transcript antisense RNA (HOTAIR) can bind to UPF1 to degrade ACSL4, thereby inhibiting ferroptosis and the impairment of neurological function in ICH mice (Jin et al., 2021).

### 5.4. Lipid peroxidation in SAH

As early as the 1980s, a team led by Asano explored the roles of lipid peroxides in SAH (Suzuki et al., 1983). They used a combination of high-performance liquid chromatography (HPLC) and gas chromatography-mass spectrometry (GC-MS) techniques to identify hydroperoxy HETEs (HPETEs) and HETEs in the cerebrospinal fluid (CSF) of SAH patients as well as healthy individuals (Suzuki et al., 1983). The results showed that compared with the average population, the content of 5-HETE in the CSF of SAH patients was significantly increased. However, the knowledge about SAH at that time led them to hypothesize that the content of this 5-HETE was related to the degree of vasospasm (Suzuki et al., 1983). Their follow-up studies also showed that 5-HETE levels were also elevated in the walls of blood vessels and the temporal cortex adjacent to SAH blood clots (Sakaki et al., 1986). Later, another group reported the up-regulation of another class of lipid peroxides, phosphatidylcholine hydroperoxide, and cholesteryl ester hydroperoxide, in the CSF of SAH patients (Kamezaki et al., 2002). However, our current knowledge indicates that neither of these two lipid peroxides mediates ferroptosis. After an extended period, there has been a lacuna in studies on lipid peroxidation in patients or animals with SAH. It is worth pointing out that 5-HETE is formed when 5-lipoxygenase catalyzes ADA (Stockwell et al., 2017). Unfortunately, no studies have investigated the mechanism of action and role of 5-lipoxygenase in SAH. In animal models of ICH and ischemic stroke, 5-lipoxygenase has been demonstrated to induce ferroptosis induction critically. 15-lipoxygenase performs a similar function as 5-lipoxygenase, only differing in the site of oxidized PUFAs (Çolakoğlu et al., 2018). It is highly expressed in microglia upon the onset of SAH. Reduction of 15-lipoxygenase level by drug application inhibits microglial ferroptosis (Gao et al., 2022). Moreover, applying its inhibitor baicalin can reduce ferroptosis after SAH and thus alleviate EBI (Zhang et al., 2020). Such lines of research on lipoxygenase may be valuable in depicting SAH pathogenesis.

Qu et al. (2021) verified the essential role of ASCL4 in ferroptosis induction in the SAH mice model for the first time. They found that the down-regulation of ACSL4 significantly inhibits lipid peroxidation and neurological damage in SAH. Two subsequent studies also examined the role of ACSL4 as a protein-level biomarker for ferroptosis (Huang et al., 2022; Yuan B. et al., 2022). The alteration of ASCL4 has higher stability than other marker molecules and has a better significance in detection. This characteristic has been confirmed in various disease models.

### 5.5. Antioxidant system in ICH

Moreover, GSH/GPX antioxidant system received attention simultaneously as more studies on lipid peroxidation were performed. In the ICH disease model, the role of GSH was first revealed by the team led by Dhandapani, who successively reported that GSH depletion leads to cell damage and death in hemin-induced astrocytes and endothelial cells (Laird et al., 2008; Sukumari-Ramesh et al., 2010). However, these forms of cell death do not accord with the features of ferroptosis. Zhang et al. (2018), for the first time, explored the expression changes and effects of GPX4 in a mouse model of ICH and showed how its levels in mouse brain tissue surrounding the hematoma were significantly reduced 2 days after ICH. Moreover, overexpression of GPX4 with adeno-associated virus significantly attenuated neuronal cell death and improved mice behavior (Zhang et al., 2018). Unfortunately, the identification of ferroptosis was not rigorous enough. In 2019, a cellular study found that selenium upregulates GPX4 by activating the transcription factors TFAP2c and Sp1 of GPX4 (Alim et al., 2019). Upregulated GPX4 can protect neurons from ferroptosis. They reported that GPX4 was upregulated in hemin-induced primary neurons, which may be related to the negative feedback regulation after ferroptosis. GSH and GPX have been repeatedly verified as critical negative regulators of oxidative stress in subsequent ICH studies. Most studies demonstrated how GSH content and the expression of GPX4 are downregulated, subject to reversing by regulating antioxidant drugs like dauricine or microRNAs to exert neuroprotective effects (Zhang et al., 2018; Peng et al., 2022).

A recent study showed in the FSP1/CoQ10 antioxidant system that the level of FSP1 was significantly reduced in the brain tissue surrounding hematomas in ICH mice, which could be treated with the drug dexpramipexole to reverse this harmful change (Wang B. et al., 2022). However, the expression pattern and the underlying mechanism with the potential role of CoQ10 have not been adequately investigated.

### 5.6. Antioxidant system in SAH

In SAH, the GSH/GPX4 antioxidant system research has been significantly more robust than in ICH, due to the early successful identification of lipid peroxides in CSF and the correlation between its content and disease prognosis (Suzuki et al., 1983; Sakaki et al., 1986). Lipid peroxidation in CSF also revealed decreased GPX activity and decreased GSH content in CSF of SAH patients (Suzuki et al., 1983). Another animal experiment showed reduced superoxide-dismutase (SOD) and GPX levels could be observed in the hippocampus of the SAH rat model (Sakaki et al., 1986). Based on such observations, the imbalance of this antioxidant system in the temporal cortex of SAH patients have also been elucidated.

Moreover, a multicenter, double-blind clinical trial showed how a seleno-organic compound, ebselen, alleviate neurological deficits in SAH patients through a GPX-like mechanism of action (Handa et al., 2000). Later, more antioxidant drugs, such as melatonin and N-acetylcysteine, upregulated the antioxidant activity of GSH/GPX, causing neuroprotection in SAH experimental animals (Ayer et al., 2008; Lu et al., 2009). Notably, any of these neuroprotective effects involve inhibition of neuronal apoptosis.

After introducing the ferroptosis concept, the main focus of the research was on a subtype of the GPX family, GPX4, which also acts as a biomarker in SAH ferroptosis. Gao et al. (2020) were the first to explore the role of GPX4 in SAH. They showed that SAH significantly reduced GPX4, which is mainly expressed in neurons, and overexpression of GPX4 alleviated lipid peroxidation-mediated cell death. Most drug-related studies also focus on the up-regulation of GPX4 and whether it can be achieved as a criterion for determining whether a drug is effective for ferroptosis treatment (Huang et al., 2022; Yuan B. et al., 2022). SLC7A11, as the critical target regulating GSH synthesis, was also found to be impaired in SAH models (Liu et al., 2022). But in terms of protein expression, the reduction is not as significant as GPX4.

A recent study also validated the inhibition of the FSP1/CoQ10 antioxidant system *in vivo* and *in vitro* SAH models. They highlighted how the activation of a well-known epigenetic regulator, Sirtuin 1 (SIRT1), alleviates neuronal ferroptosis in SAH by upregulating the expression of FSP1 and CoQ10B (Yuan B. et al., 2022). Their finding adds critically to the existing knowledge about the antioxidant mechanism underlying ferroptosis after a hemorrhagic stroke and deserves more investment in future research.

## 6. The research dilemma and prospects of ferroptosis

### 6.1. Technical barrier of ferroptosis marker detection

Apoptosis, pyroptosis, and autophagy involve hallmark molecules that can execute the cell death program, enabling researchers to detect these PCDs by simple but specific techniques (Fang et al., 2020). In addition, technical means such as TUNNEL staining and annexin v-pi staining help to locate the apoptotic cells (Luchetti et al., 2023). For autophagy, the autophagic-lysosome is its identification marker (Rodgers et al., 2022). However, the currently established markers for ferroptosis, GPX4, SLC7A11, ACSL4, and several iron metabolism-related indicators fail to point to ferroptosis directly. In 2017, two independent groups established that PEs-AA/ADA-OOH are the direct executor of ferroptosis, providing a novel yet convincing biomarker for validating ferroptosis (Kagan et al., 2017; Wenzel et al., 2017). However, separating and purifying this specific phospholipid is challenging as the tedious and elaborate process involves high-performance liquid chromatography and mass spectrometry with high-purity standards for comparison. Very few laboratories worldwide accurately detect PEs-AA/ADA-OOH. Hence minimal testing has been performed to evaluate its content in brain tissue after a hemorrhagic stroke. Moreover, no ferroptosis-specific tissue staining techniques or flow cytometry have been developed. Therefore, the identification of ferroptosis still involves evaluating morphological changes observed by TEM and verifying many biomarkers.

### 6.2. The diversity of ferroptotic cell types

Evidence from most of the current studies suggests that the cell types undergoing ferroptosis after hemorrhagic stroke mainly involve neurons (Zhang et al., 2018; Gao et al., 2020; Huang et al., 2022). The recovery of neural function is largely due to the repair of neurons. Moreover, the primary regulators of ferroptosis, ACSL4, and GPX4, were mainly localized in neurons, further validating the hypothesis that ferroptosis occurs in neurons after an injury. Mitochondrial ferroptotic damage within neurons under TEM observation is critical for identification. However, multiple other cell types in the brain have also been reported to undergo ferroptosis.

Several studies have reported the role of ferroptosis in microglia, which put forward by Kapralov et al.’s (2020) research on macrophage ferroptosis. They found M2-type macrophages to be more sensitive than M1-type macrophages to ferroptosis. Studies have shown that ferroptosis is prevalent in SAH microglia, and the sensitivity of M1 and M2 microglia to ferroptosis is also similar to that of peripheral macrophages (Wu et al., 2020). Two subsequent studies indicated that this sensitivity of microglia to ferroptosis may be regulated by the expression of intracellular NRF2 or iNOS (Cui et al., 2021b; Qu et al., 2022). Nevertheless, the specific role of microglia upon the onset of ferroptosis has not been fully explored.

Li et al. (2018) used TEM to observe mitochondria characteristics of ferroptosis in axons of oligodendrocytes. Subsequently, they further systematically presented ample evidence of ferroptosis in oligodendrocytes after ICH (Shen et al., 2022). Dexpramipexole has also been reported to attenuate white matter damage caused by oligodendrocyte ferroptosis in mice after ICH, thereby improving spatial localization and motor function (Wang B. et al., 2022). Moreover, ferroptosis has also been reported to be involved in white matter damage after spinal cord injury, which can be suppressed by the application of Fer-1 (Chen et al., 2022).

In an Alzheimer’s disease study, ferroptosis was shown to occur in astrocytes and is positively regulated by NADPH oxidase 4-mediated impairment of mitochondrial metabolism (Park et al., 2021). Another study identified ferroptosis in cultured astrocytes. Although the validation evidence for ferroptosis in this *in vitro* experiment is not comprehensive (Li S. et al., 2021), it still provides an outlook for the existence of ferroptosis in astrocytes.

In addition, some studies or reviews have proposed the feasibility and essential role of ferroptosis in endothelial cells (Qin et al., 2021). However, evidence for it, especially in the central nervous system (CNS), still appears to be lacking. In summary, studying the ferroptosis of cells other than neurons holds great promise in the CNS. The lack of practical means makes this line of research somewhat challenging to identify the specific ferroptotic cell localization. We have presented the current studies for various types of ferroptosis in the CNS in Table 1.

**TABLE 1:** Ferroptosis in different cell types in the central nervous system (CNS).

Disease model	Cell type	Means for identification	Potential mechanisms	References
Collagenase-induced mouse model of ICH; Hemin-induced primary cortical neurons.	Neuron	***In vivo:*** FJB staining; Measurement of eicosanoids; Metabolic analysis of lipoxygenase. ***In vitro:*** Adenoviral overexpression of antioxidant enzymes; Measurement of eicosanoids.	Neuronal ferroptosis may be involved in neurological impairment after ICH.	Karuppagounder et al., 2018
Collagenase-induced mouse model of ICH; Hb/ferrous iron-induced OHSCs.		***In vivo:*** FJB/FJC staining; PI staining; TEM; Perls’ staining; Biomarker analysis (COX-2; GPX-4); 4-HNE and MDA measurement. ***In vitro***: DIPY 581/591 C11; Biomarker analysis.	Neuronal ferroptosis may contribute to neurological defects after ICH; Fer-1 treatment can alleviate neuronal ferroptosis.	Li et al., 2017a
Endovascular perforation mouse model of SAH; Hemin-induced HT22 cells.		***In vivo:*** FJC staining; GSH, GPX4, MDA measurement; TEM; Biomarker analysis (ACSL4; GPX-4; xCT; COX-2). ***In vitro:*** Cell viability analysis; Mitochondrial functions evaluation; DIPY 581/591 C11.	Neuronal ferroptosis may contribute to neurological defects after SAH; Lip-1 treatment can alleviate neuronal ferroptosis.	Cao et al., 2021
Endovascular perforation rat model of SAH; Oxyhemoglobin-induced SH-SY5Y cells.		***In vivo:*** FJC staining; GSH and GPX4 activity measurement; TEM; Biomarker analysis (GPX-4; FPN; TfR1); Iron stain assay. ***In vitro:*** Cell viability analysis; DIPY 581/591 C11.	Neuronal ferroptosis may contribute to neurological defects after SAH; Fer-1 treatment can alleviate neuronal ferroptosis.	Li Y. et al., 2021
Endovascular perforation mouse model of SAH; Hemin-induced BV2 cells.	Microglia	***In vivo:*** GSH, GPX4 activity, and MDA measurement; TEM; Biomarker analysis (ALOX-15; GPX-4; xCT); ***In vitro:*** Cell viability analysis; DIPY 581/591 C11.	M2-type microglia may be more sensitive to ferroptosis after SAH than M1-type microglia.	Gao et al., 2022
I.p. injection of LPS -induced inflammation model; LPS-induced primary neonatal microglia.		***In vivo:*** DCFH-DA for intracellular ROS; MDA measurement. ***In vitro:*** Cell viability analysis; LDH release assay; DIPY 581/591 C11.	RSL3 inhibited LPS-induced inflammation of microglia in a Nrf2-dependent way.	Cui et al., 2021b
Endovascular perforation rat model of SAH; Oxyhemoglobin-induced BV2 microglia.		***In vivo:*** FJB staining; MDA measurement; Biomarker analysis (GPX-4; FPN; Tf; xCT; TfR1; COX-2). ***In vitro:*** Cell viability analysis; DIPY 581/591 C11.	Upregulated iNOS may mediate the defense effect of M1-type microglia against ferroptosis after SAH.	Qu et al., 2022
I.v. injection of autologous blood induced IVH model; Hemin induced primary OPCs.	Oligodendrocyte progenitor cells	***In vivo:*** PI staining; Perls’ staining; TEM; Biomarker analysis (GPX-4; xCT; COX-2); ***In vitro:*** GSH and GPX4 activity measurement; DIPY 581/591 C11.	Ferroptosis is the primary cell death form in Hemin-induced and hemorrhagic stroke-induced OPCs death.	Shen et al., 2022
Autologous blood-induced mouse model of ICH.	Oligodendrocytes	***In vivo:*** Dihydroethidium staining for ROS identification; Perls’ staining; MDA measurement; TEM; Biomarker analysis (FSP1; GPX-4).	Some degree of ferroptosis in oligodendrocytes after ICH, which can be alleviated by treatment with dexpramipexole.	Wang B. et al., 2022
RSL-3 induced ONL-93 oligodendrocytes.		***In vitro:*** PI staining; Cell viability analysis; ROS identification; Biomarker analysis (ACSL4; xCT; GPX4; FSP1); GSH, GPX4 activity, and MDA measurement; Mitochondrial lipid peroxidation detection.	Lip-1 can inhibit RSL-3-induced ferroptosis in ONL-93 oligodendrocytes by upregulating GPX4.	Jhelum et al., 2020
*Mbp* knockout *Shiverer* mice; Primary Oligodendrocytes from Pelizaeus-Merzbacher patient.		***In vivo:*** Click-iT^®^ lipid peroxidation detection; TEM; CellROX^®^ ROS sensor; Biomarker analysis (GPX-4; FPN; Tf; xCT; TfR1). *In vitro*: CellROX^®^ ROS sensor; Biomarker analysis.	Iron Chelation can reduce oligodendrocyte ferroptosis in Pelizaeus-Merzbacher disease.	Nobuta et al., 2019
APP/PS1 double-transgenic mice	Astrocyte	***In vivo:*** Mitochondrial ROS analysis; GSH, the ratio of GSH/GSSG and GSSG	Astrocyte ferroptosis in Alzheimer’s disease is promoted by upregulated NADPH oxidase 4.	Park et al., 2021
model of AD.		measurement; glutamate-cysteine ligase catalytic subunit measurement.	
Angiotensin II-induced primary astrocyte.		***In vitro:*** Cell viability analysis; Biomarker analysis (GPX-4; Nrf2; HO-1; COX-2); LDH release assay; Intracellular total iron assay.	Fer-1 reduced Angiotensin II-induced ferroptosis in astrocytes.	Li S. et al., 2021
High glucose-induced human retinal capillary endothelial cells (HRCECs).	Endothelial cells	***In vitro:*** Cell viability analysis; PI staining; SOD, GPX4 activity, and MDA measurement; Biomarker analysis (GPX-4); DIPY 581/591 C11.	High glucose-induced HRCECs ferroptosis can be facilitated by TRIM46-induced GPX4 ubiquitination.	Qin et al., 2021

### 6.3. Uncertainty about the magnitude and duration of ferroptotic action

Developing clinically effective drugs against ferroptosis is a crucial goal of our research. The elucidation of the molecular mechanism of ferroptosis after hemorrhagic new stroke will facilitate the development of novel and more efficacious drugs. There is electron microscopic evidence that ferrotropis existed earlier in ICH than necrossis and autophagy (Li et al., 2018). However, a significant challenge in this area of research involves the failure to define the contribution of ferroptosis to neurological impairment after hemorrhagic stroke and the difficulty of determining which specific period after hemorrhagic stroke plays a vital role in the ferroptosis effect. The crucial questions remain: faced with various PCDs after injury, which one aspect of research should we focus on? Is it possible to target in combination some of these forms of death to achieve the highest therapeutic effect? Are the dominant PCDs different across periods after hemorrhagic stroke? In fact, a large amount of basic studies related to ferroptosis are lack of reliable experimental evidence. For example, many articles have not provided evidence of TEM, or arbitrarily concluded that ferroptosis changes only through the changes in the end products of lipid peroxidation. And most of studies have not been repeatedly verified or even can’t be reproduced at all. It has to be admitted that in view of the above problems, there is no treatment based on iron death that can be carried out in a phase I clinical trial. These issues are of paramount importance to be addressed in the clinical translation of preclinical drug research for hemorrhagic stroke. In the face of the above problems, using single cell sequencing technology to classify dying cells in biology may be a direction worth trying. Combining with the pseudotime analysis, we may classify the outcomes of these cells by detecting the marker molecules of PCDs, so as to solve the difficult problems of time point after stroke and proportion of ferroptosis in PCDs.

## Author contributions

FP and CW designed and wrote the manuscript. WX and JD contributed to the literature searches and analyses. FP and WX critically revised the manuscript. All authors approved the final manuscript.
